# Nonlinear Landau Fan Diagram for Graphene Electrons Exposed to a Moiré Potential

**DOI:** 10.1021/acs.nanolett.3c04444

**Published:** 2024-02-02

**Authors:** Pilkyung Moon, Youngwook Kim, Mikito Koshino, Takashi Taniguchi, Kenji Watanabe, Jurgen H. Smet

**Affiliations:** † Arts and Sciences, NYU Shanghai, Shanghai 200124, China; ‡ NYU-ECNU Institute of Physics at NYU Shanghai, Shanghai 200062, China; § Max-Planck-Institut für Festköperforschung, Stuttgart 70569, Germany; ∥ Department of Physics and Chemistry, DGIST, Daegu 42988, Korea; ⊥ Department of Physics, Osaka University, Toyonaka 560-0043, Japan; # International Center for Materials Nanoarchitectonics, National Institute for Materials Science, Tsukuba 305-0044, Japan; 7 Research Center for Functional Materials, National Institute for Materials Science, Tsukuba 305-0044, Japan

**Keywords:** graphene, moiré superlattice, quantum Hall effect, nonlinear Landau fan

## Abstract

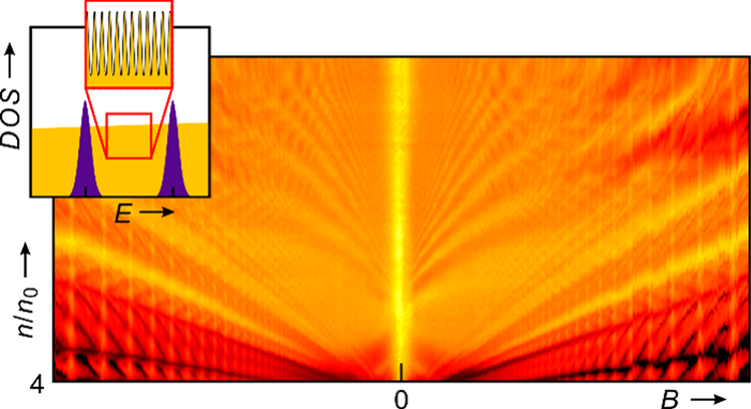

Due to Landau quantization, the conductance of two-dimensional electrons exposed to a perpendicular magnetic field exhibits oscillations that generate a fan of linear trajectories when plotted in the parameter space spanned by density and field. This fan looks identical, irrespective of the dispersion and field dependence of the Landau level energy. This is no surprise because the position of conductance minima depends solely on the level degeneracy that is linear in flux. The fractal energy spectrum that emerges within each Landau band when electrons are also exposed to a two-dimensional superlattice potential produces numerous additional oscillations, but they also create just linear fans for identical reasons. Here, we report conductance oscillations of graphene electrons exposed to a moiré potential that defy this general rule and form nonlinear trajectories in the density-field plane. We attribute this anomalous behavior to the simultaneous occupation of multiple minibands and magnetic breakdown-induced open orbits.

The spectral gaps in the density of states (DOS) that originate from Landau quantization of the spectrum of a two-dimensional (2D) electron system exposed to a perpendicular magnetic field *B* produce a fan of linear trajectories in a plot of the transport quantities in the parameter plane spanned by electron density *n* and *B*.^1^ The linear trajectories are described by a Diophantine equation of the form *n*/*n*_0_ = *t* × φ/φ_0_ + *s*, where φ/φ_0_ is the normalized magnetic flux per unit cell and *n*/*n*_0_ is the density normalized to total density *n*_0_ that can be accommodated by the partially filled band, ignoring any extra degeneracies such as spin and valley degrees of freedom. Integers *s* and *t* are topological integers representing the band filled at zero field and the quantized Hall conductivity, respectively.^2−5^ This outcome is independent of the size and *B* dependence of the gaps because only the level of degeneracy matters and just depends on the number of magnetic flux quanta that pierce the system. Hence, it is not possible to extract any information about the energy spectrum such as the Landau level energy spacing from such a (*n*, *B*) or Wannier diagram. To obtain the gap sizes, we need to perform either cyclotron resonance or thermal activation studies measuring the required thermal energy to overcome the gap closest to the chemical potential.

When 2D electrons are subjected to a 2D superlattice potential, Bragg scattering and zone folding convert the original bands into a series of much smaller minibands.^6^ Each miniband can host a density *n*_0_ = 1/*A*, where *A* is the area of the unit cell or an integer multiple thereof, if carriers have additional degrees of freedom. This *n*_0_ can be much smaller than the density that a conventional crystal band holds, and multiple minibands can easily be filled through gating. Additional linear Landau fans emanate whenever a miniband is completely filled or emptied. In addition, the superlattice potential broadens these Landau levels into bands that themselves develop internal subbands. This results in a fractal spectrum within each Landau band that is termed Hofstadter’s butterfly. The additional gaps separating these subbands produce Brown–Zak oscillations and generate linear trajectories in the (*n*, *B*) plane that are again described by a Diophantine equation.^1−5,7−13^ As before, it is not possible to extract energy spacings because the conductance minima appear at densities and fields determined by the degeneracy of the states unrelated to the gap size.

van der Waals stacking of 2D materials offers unprecedented control in creating such devices where electrons are exposed to a moiré interference potential with a periodicity of a few to a few tens of nanometers.^2−5,8−15^ Here, we have investigated graphene electrons that are subjected to the moiré potential created by an hBN layer aligned with the graphene lattice. While the magnetotransport features in a Wannier diagram are indeed dominated by linear trajectories formed by conductance minima due to Landau quantization of the minibands as well as the internal fractal Landau band structure, unanticipated nonlinear trajectories that do not fulfill a flux linear Diophantine equation are also observed. This anomalous behavior appears in the density regime where more than just one miniband is partially occupied and the Fermi surface areas that these partially filled minibands contribute are very different. We demonstrate that these nonlinear trajectories in the (*n*, *B*) plane offer the unique opportunity to extract information about the spectral gaps. This technique should be applicable to a broader class of systems, such as twisted bilayer and multilayer graphene with various stacking configurations.

Here, magnetoresistance data were recorded on two hBN-encapsulated monolayer graphene devices, D1 and D2. In both, the top hBN layer was approximately aligned with the graphene, generating a moiré potential with a period of ∼13.9 nm (section S2 of the Supporting Information). The second hBN layer at the bottom was intentionally misaligned to avoid another moiré superlattice. The doped silicon substrate served as the back gate in D1, while a graphite layer was used as the top and bottom gate in D2. Because the transport behavior is consistent, we show results from only D1 in the main text. Data acquired on D2 are available in section S3. Details of the fabrication can be found in Methods. The bottom parts of panels a and b of Figure 1 display longitudinal resistance *R*_*xx*_ at *B* = 0 and Hall resistance *R*_*xy*_ at 0.2 T as a function of *n*/*n*_0_. Also shown are the electronic band structure (Figure 1c) along the high-symmetry points of the superlattice Brillouin zone (section S4) and density of states (Figure 1d) calculated using the effective continuum model for a heterostructure with a 0° twist.^16^ As anticipated for electrons exposed to a moiré potential, *R*_*xx*_ exhibits three peaks. The maxima at *n* = ±4*n*_0_ signal full occupation or depletion of the lowest conduction and valence minibands. The factor of 4 accounts for the spin and valley degrees of freedom. The resistance peak at *n* = 0 corresponds to the main charge neutrality point (CNP) and is a result of the vanishing DOS as the chemical potential approaches the Dirac point. The sign reversal of *R*_*xy*_ at these three densities is consistent with this interpretation. Because Bragg scattering of the superlattice produces a van Hove singularity on either side of the main CNP (Figure 1c,d), an additional sign reversal of *R*_*xy*_ occurs due to a Lifshitz transition, i.e., an abrupt change in the Fermi surface topology, when the chemical potential crosses these singularities.^17,18^ The excellent agreement between the experiment and theory proves that electrons are subjected to the hBN-induced moiré superlattice.

**Figure 1:**
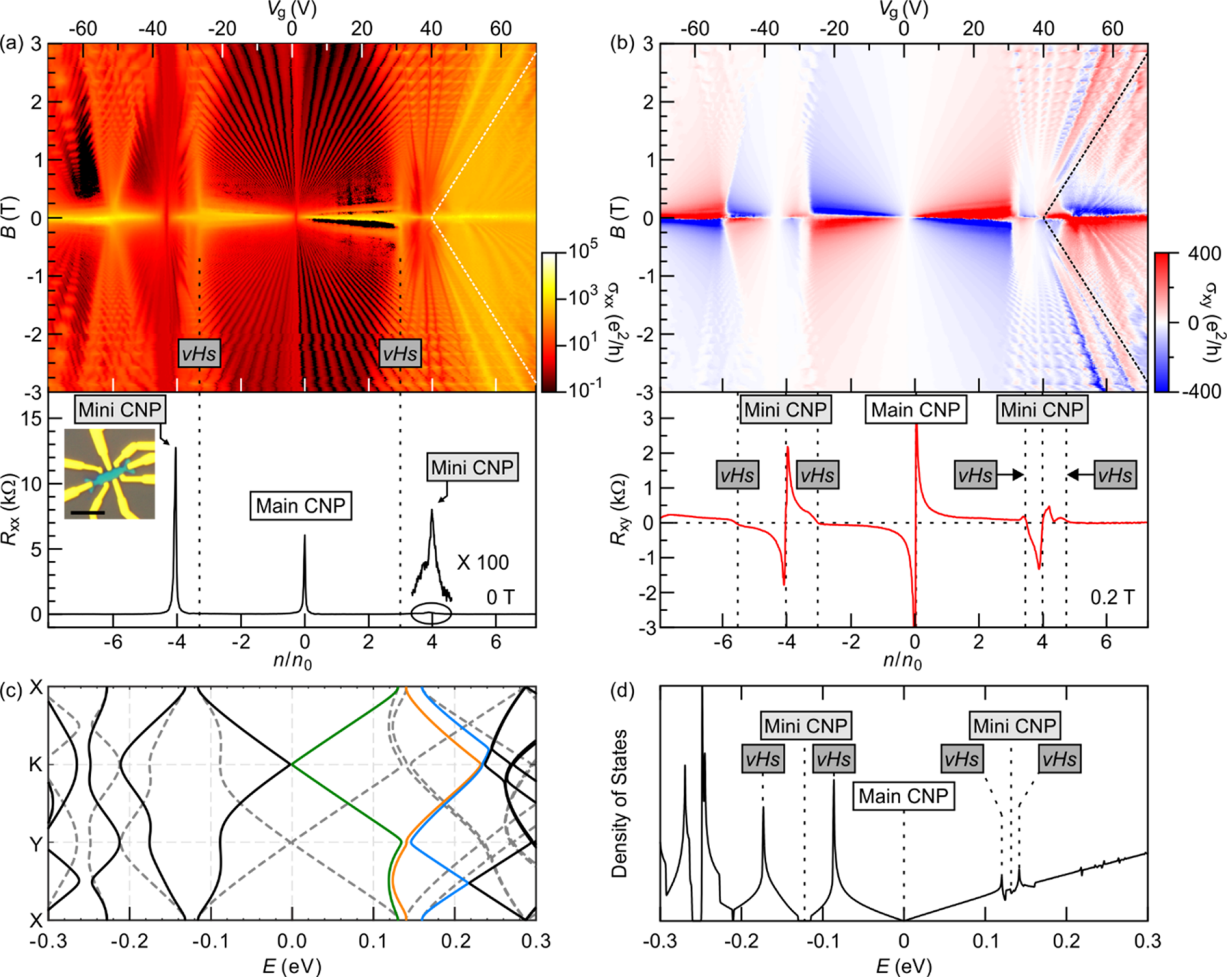
Magnetotransport of a graphene/hBN heterostructure with a 0° twist angle. (a) σ_*xx*_ as a function of gate voltage (*V*_g_) or normalized density (*n*/*n*_0_) and magnetic field (*B*) for fields up to ±3 T (top). *V*_g_ and *B* are increased in 5 mV and 10 mT steps, respectively. The data are recorded at ∼30 mK. The color scale is logarithmic. Dashed lines mark the location where the chemical potential crosses a van Hove singularity in the lowest conductance and valence minibands. On the right of the white dotted lines, conductivity features trace nonlinear trajectories. *R*_*xx*_ as a function of *n*/*n*_0_ for *B* = 0 T (bottom). The inset shows an image of the device. The scale bar corresponds to 5 μm. (b) Similar to panel a but for σ_*xy*_ = *R*_*xy*_/{[*R*_*xx*_(*w*/*L*)]^2^ + *R*_*xy*_^2^} × *R*_K_ (top). The region with Hall conductivity features that form nonlinear trajectories is to the right of the dotted black lines. Single-line trace of *R*_*xy*_ at *B* = 0.2 T (bottom). The horizontal dotted line corresponds to *R*_*xy*_ = 0 Ω, and the vertical dotted lines mark zero crossing or charge inversion points. Charge inversion occurs when the chemical potential reaches the Dirac point (main CNP), a band edge (mini CNP), or a van Hove singularity (vHs). (c) Band dispersions along the high-symmetry points of Figure S3 for the graphene/hBN heterostructure with a 0° twist angle. Solid (dotted) lines represent the bands near the K (K′) valley of the monolayer graphene. Green, orange, and blue highlight the dispersion of the first, second, and third conduction minibands, respectively. (d) Density of states plotted vs electron energy.

The top graphs in panels a and b of Figure 1 show color maps of longitudinal conductivity σ_*xx*_ and Hall conductivity σ_*xy*_ as a function of back gate voltage *V*_g_ or *n*/*n*_0_ and *B*. The conductivities are obtained from the inversion of the resistance tensor: σ_*xx*_ = *R*_*xx*_(*w*/*L*)/{[*R*_*xx*_(*w*/*L*)]^2^ + *R*_*xy*_^2^} × *R*_K_ and σ_*xy*_ = *R*_*xy*_/{[*R*_*xx*_(*w*/*L*)]^2^ + *R*_*xy*_^2^} × *R*_K_, where *w* and *L* are the sample width and length, respectively, and *R*_K_ is the von Klitzing constant (≈25 812 Ω). Minima in σ_*xx*_, which track the spectral gaps, are visible down to 0.8 T. They appear as straight lines in this Wannier diagram and follow the Diophantine equation. They converge to the main CNP at zero energy or to the top or bottom of the miniband, termed mini CNPs, as *B* approaches zero. A portion of the experimental data in Figure 1a is replotted in Figure 2a with an optimized color scale near *n*/*n*_0_ = 4 (*V*_g_ ≈ 40 V) to better distinguish this. A color map of the conductivity that extends to higher fields is included in section S1. It reveals the Hofstadter spectrum.^7^

**Figure 2:**
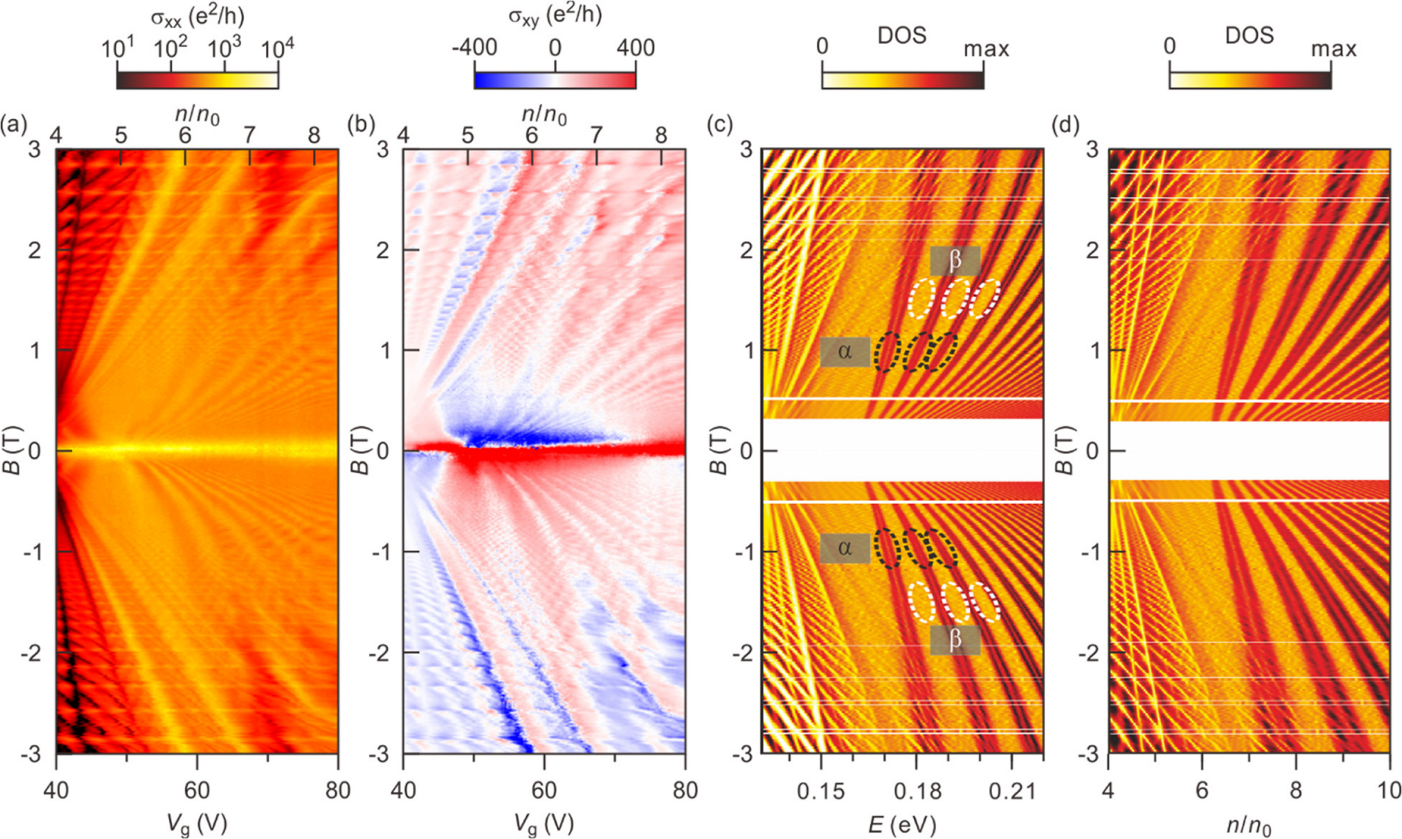
Comparison between the magnetotransport properties and the density of states of the graphene/hBN heterostructure with a 0° twist angle. (a) σ_*xx*_ measured as a function of *B*, *V*_g_, and *n*/*n*_0_. (b) Same as panel a but for σ_*xy*_. (c) Theoretically calculated DOS in the *E*–*B* plane. The ellipses demarcated with white dashed lines are regions where only the β orbits contribute to the DOS. These orbits produce a nearly continuous density of states, resulting in the bright orange background. Isolated discrete levels cannot be resolved. In contrast, the ellipses demarcated with dark dotted lines mark regions where the DOS is strongly enhanced by quantization of the closed α orbits. These do produce well-separated Landau levels with finite energy spacing. Three such Landau levels are marked with the dark ellipses. (d) Same as panel c but in the *n*/*n*_0_–*B* plane.

The features in the Wannier diagram discussed above have been addressed previously in the literature.^2−5^ In this work, we focus on an unusual sequence of minima in σ_*xx*_ that appears at weak fields (*B* < 3 T) when more than just the lowest conduction miniband is occupied (*n*/*n*_0_ > 5 or *V*_g_ > 50 V). In Figure 1a and panels a and b of Figure 2 (40 V < *V*_g_ < 80 V), the minima in this regime trace trajectories that are distinct from the common linear Landau fan. The trajectories are not linear in the (*n*, *B*) plane, but parabolic-like, and they do not converge to one of the nearby band edges at *n*/*n*_0_ = 4 or 8 (*V*_g_ = 40 or 80 V) when *B* → 0. Note the overall high value of conductivity (Figure 2a), exceeding 100 e^2^/h, even at the minima. This is much higher than that for the previously discussed minima associated with either Landau quantization or internal Landau band gaps. These nonlinear features weaken as *B* is increased. They disappear near 3 T. Section S3 contains a data set for D2, and similar nonlinear trajectories appear for the same density range. Because within a relaxation time approximation the conductivity is approximately proportional to the DOS, it should come as no surprise that a calculation of the latter for this aligned graphene/hBN superlattice shows strong similarities to the conductivity map in Figure 2a. Panels c and d of Figure 2 display a color rendering of the DOS as a function of *B* and *E* and as a function of *B* and *n*/*n*_0_, respectively. For the sake of simplicity, a Gaussian broadening with a constant full width at half-maximum of 0.5 meV was applied. The resemblance of the experimental data for *n*/*n*_0_ > 5 is striking. Figure 2c shows that this energy range is filled with nearly continuous states (bright orange background marked by β) as well as Landau levels with finite energy spacing (dark orange lines marked by α). In the (*n*, *B*) plane (Figure 2d), the DOS maxima (dark orange lines), or the minima between them, exhibit nonlinear trajectories and do not converge to *n*/*n*_0_ = 4 or 8 as *B* → 0, consistent with experiment. Plots of the DOS that extend to a wider range of electron energy and density clearly show the linear and nonlinear trajectories (section S5).

To identify the origin of the nonlinear features in the conductivity and the DOS map, studying the isoenergetic contours at different Fermi energies *E*_F_ is instructive. The top panel of Figure 3a shows the dispersion of the first three conduction minibands near the K symmetry point for *B* = 0 calculated with the effective continuum model^16^ (section S6). These minibands are not separated in energy. While the first miniband (green) only slightly overlaps with the second (orange), the second miniband overlaps with the third miniband over an extended range of energies when *E* > 0.15 eV. This is also apparent from the dispersions of these minibands along the high-symmetry points in Figure 1c, where these minibands were color-coded in the same fashion as in Figure 3a. The isoenergetic contours are plotted for six different *E*_F_ values in Figure 3b. When *E* > 0.15 eV, the third miniband develops pockets centered around the X and Y symmetry points of the reduced Brillouin zone. The contours encircling an X symmetry point (purple shaded lines in the panel with *E* = 0.18 eV) are well separated in reciprocal space from the isoenergetic contours of the second miniband. The quantization of the corresponding real space orbital in a *B* field should therefore produce a set of discrete Landau levels. This is the origin of the Landau levels that we can identify by the dark orange lines α in Figure 2c. In the remainder, we refer to these orbits as α orbits (purple shaded lines in Figure 3b). The contours encompassing a Y symmetry point, on the contrary, nearly touch the isoenergetic contours of the second miniband. Under these circumstances, electrons have a finite probability of tunneling between this orbital of the third miniband and the orbital associated with the second miniband (yellow shaded lines in the panel with *E* = 0.18 eV). In general, as *B* increases, the real space orbits shrink. Hence, the uncertainty in real space decreases at the expense of an increased uncertainty in momentum space. The latter scales proportional to  and enables tunneling. This effect, well-known as magnetic breakdown,^19^ effectively converts both orbits involved into open snake orbits, which we term β orbits hereafter (along the yellow shaded lines in Figure 3b; one snake orbit running in the vertical direction is highlighted with a dotted line and arrows). These open β orbits do not contribute well-separated Landau levels but produce a nearly continuous contribution to the spectrum. They contribute the bright orange background marked as β to the DOS in Figure 2c. Panels e and f of Figure 4 summarize schematically how the energy spectrum and density of states look in this regime where magnetic breakdown occurs. The schematic energy spectrum is composed of well-separated Landau levels in purple with energies  as created by Landau quantization of the real space α orbits as well as narrowly spaced levels marked in yellow that stem from the β orbits. In the DOS, all Landau levels are broadened with a Gaussian so that the yellow levels make up a nearly continuous background. Interestingly, α and β orbits virtually do not mix even at higher energies, which is apparent from the simple crossing of these energy contours encircling the X and Y symmetry points at *E* = 0.23 eV; they persist and do not interfere almost up to *n*/*n*_0_ = 12 or upon complete filling of the third miniband. Similar intersecting Landau levels have been observed in a system with a scalar potential.^20^

**Figure 3:**
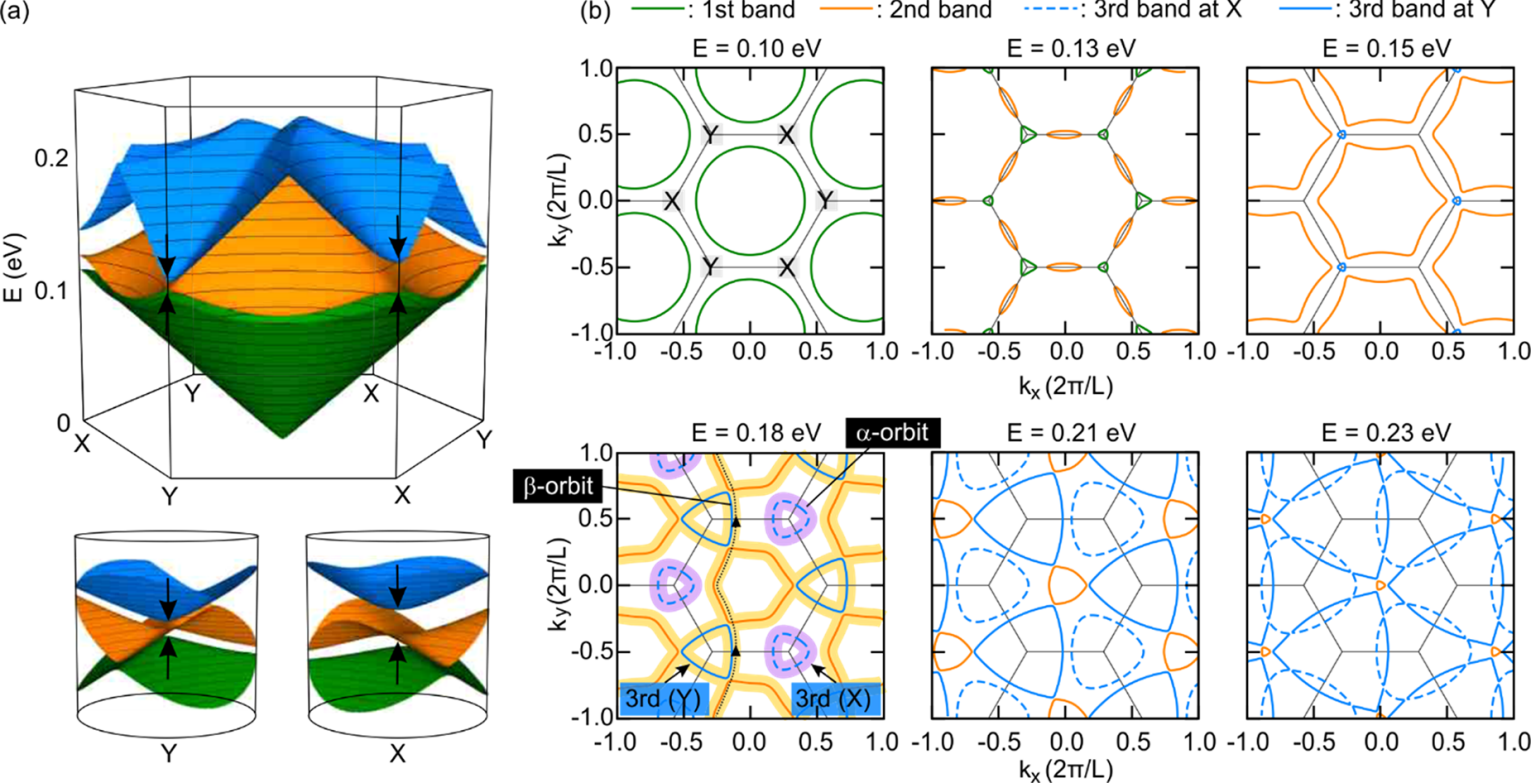
(a) Three-dimensional plot of the first three conduction minibands centered at the K symmetry point of the reduced Brillouin zone (Figure S3) and calculated with the effective continuum model (top). Band dispersion of the three bands in the top panel near the X and Y corners (bottom) (eq S4 of the Supporting Information). (b) Energy contours of the first three conduction minibands at six different Fermi energies. The green, orange, and blue lines show the contours of the first, second, and third minibands, respectively. The pockets of the third miniband centered around X (blue dashed lines) are bounded by α orbits (purple shaded lines in the panel with an *E* of 0.18 eV). These orbits are well separated in reciprocal space from the other isoenergetic contours and almost do not interact with them. The third miniband also has a second set of electron pockets centered around Y (blue solid lines). The bounding orbit in reciprocal space hybridizes with the Fermi surface of the second miniband in the presence of a magnetic field via magnetic breakdown (yellow shaded lines in the panel with an *E* of 0.18 eV). This produces a hybrid, snakelike orbit with a much larger area in reciprocal space (β orbit). One such snake orbit along *k_y_* is marked with a dotted line and two arrows along its path.

**Figure 4:**
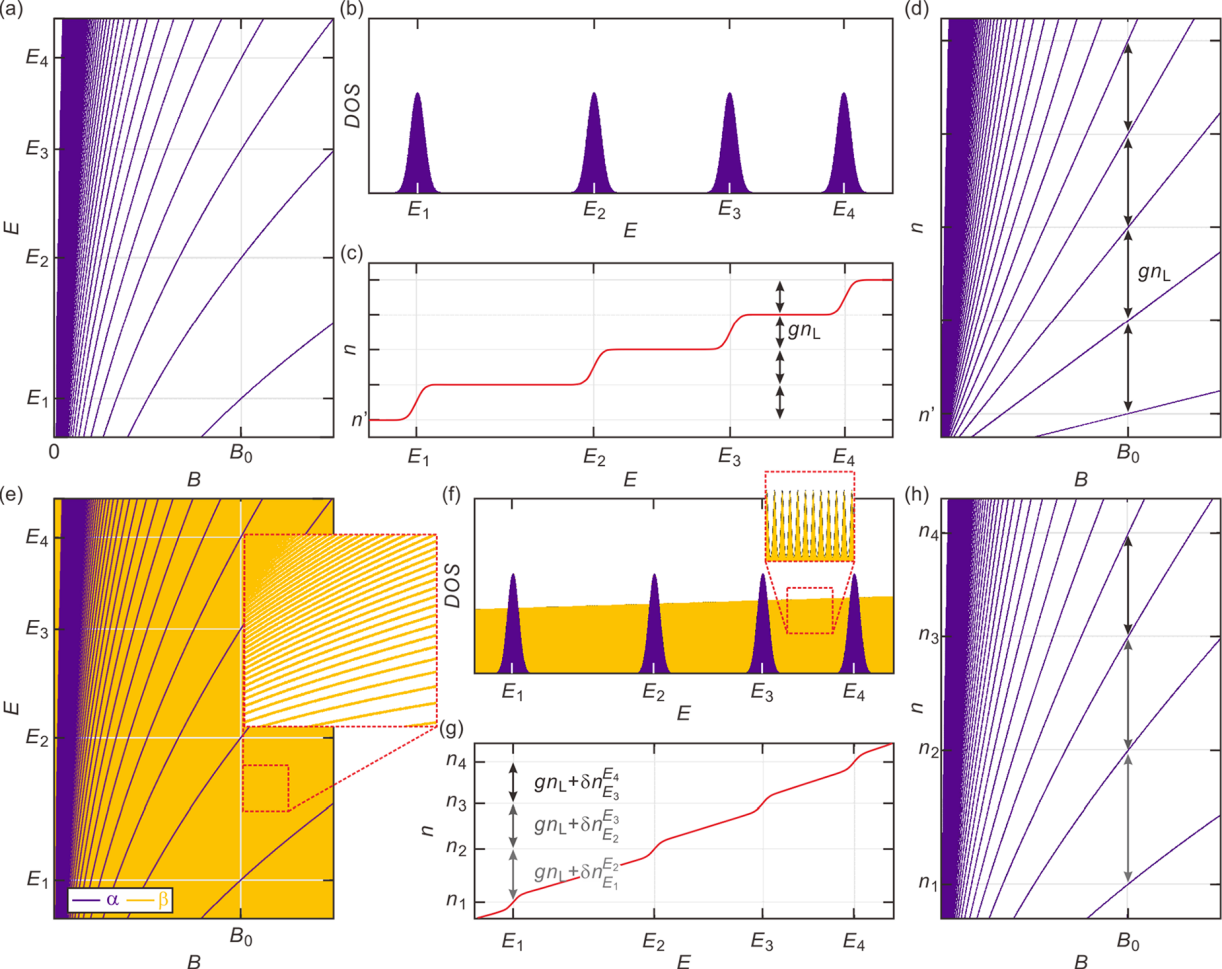
(a–d) Schematic energy spectra and Wannier diagrams of a system with a single set of Landau levels α. For illustrative purposes, the Landau level spectrum of an unproximitized graphene layer is used, although any arbitrary spectrum that is non-equidistant and exhibits level energies that depend in a nonlinear fashion on field can be chosen and would still lead to the same linear Wannier level diagram. (a) Landau levels in *E*–*B* space. *E*_*i*_ (*i* = 1, 2, 3, or 4) represents the energy of the Landau levels at a field *B*_0_. (b) Density of states and (c) electron density *n* of the series of Landau levels in panel a at a field *B*_0_ plotted vs *E*. (d) Wannier diagram in *n*–*B* space. Note that regardless of the energy spacing between the levels, *E*_*i*_, in panel b, the change in the electron density to populate the next level is always the same, *gn*_L_. Here, *n*_L_ = *eB*/*h* is the degeneracy of each Landau level without counting other degrees of freedom and *g* is the degeneracy of the bands due to, for instance, spin and valley degrees of freedom. (e–h) Plots similar to panels a–d, respectively, but for a system with two sets of Landau levels α and β with very different energy spacing. Note that the electron density occupying β orbits between  and  () depends on energy difference  (g). Hence, the energy level spacing affects the level trajectories in the Wannier diagram (h).

The coexistence of the nearly continuous spectrum from the β orbits with a spectrum of well-separated Landau levels from the α orbits is at the core of the observed nonlinear features in the Wannier diagram. To corroborate this assertion, we derive the Wannier diagram for a spectrum as described above and compare it with a system that has a spectrum composed of just a single Landau level sequence (Figure 4a,b). In general, Landau level energies are not necessarily equidistant or linear in *B*. The density to completely fill an extra level in Figure 4b depends solely on the degeneracy, which, irrespective of the band structure details, is equal to the number of flux quanta that thread through the sample (Figure 4c). Despite the lack of equidistant spacing and nonlinear *B* dependence, they appear to be equidistant (Diophantine equation, Figure 4d) in the (*n*, *B*) plane and generate conductivity minima that form linear trajectories in the Wannier diagram. For the spectrum shown in Figure 4e, on the contrary, the change in density, Δ*n*, required to fill the next α orbit level is no longer solely determined by its degeneracy, because additional states of the nearly continuous β orbit spectrum need to be filled. The Δ*n* required to increase the chemical potential from the peak in the DOS at  to the neighboring peak at  equals
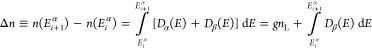
1where *n*(*E*) is the total density at energy *E*, *D*_*α*_ and *D*_*β*_ represent the DOS for the α and β orbits, respectively, *n*_L_ = *eB*/*h* is the degeneracy of a single Landau level, and *g* is the additional degeneracy due to degrees of freedom other than the orbital one. When *D*_*β*_(*E*) equals zero, the spectrum is composed of only a single series of Landau levels; eq 1 then just yields *gn*_L_, and full Landau level occupation generates the usual linear Landau fan in the *n*–*B* plane, independent of central energies  as shown in Figure 4d. For a non-zero *D*_*β*_(*E*), however, the last term in eq 1 becomes relevant and the information on the energy spacing between the α orbit Landau levels is embedded in Δ*n*. Full occupation of α orbit levels yields nonlinear trajectories (see Figure 4h), if  – , *D*_*β*_, or both are nonlinear in field. We note that panels d and h highlight conductivity maxima, because  and  refer to adjacent maxima in the DOS. In principle, we can use eq 1 also for conductivity minima by changing the integration limits to energies between the α levels. Hereafter, we will follow the conductivity maxima to directly trace . Provided *D*_*β*_ is known or can be estimated, we can reverse engineer the α orbit level spacing from the Wannier diagram. No assumptions or knowledge of *D*_*α*_ are required. In other words, only knowledge of *D*_*β*_ is needed to determine the energy spacing . Such reverse engineering is not possible in a system with a single series of discrete Landau levels, because then *D*_*β*_(*E*) = 0. If *D*_*β*_(*E*) slowly varies with energy, we can obtain the level spacing from
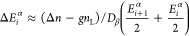
2a formula that indeed works only when . In principle, we can use eq 2 to probe the Landau level spectrum of any 2D material proximitized with a reference material with a much smaller level spacing and a known density of states (e.g., metal, “case A”) or we can extract the Landau level spectrum in a system having two different series of Landau levels in the same energy range but with very different level spacing (“case B”).

With this background and eq 2, it is possible to extract the level spectrum generated by the α orbits from the data. Because our heterostructure corresponds to case B, we need to obtain *D*_*β*_(*E*) before using eq 2. For electron population, the total DOS, *D*_*α*_(*E*) + *D*_*β*_(*E*), is close to that of an unproximitized graphene monolayer. This approximation is justified, because the dispersion for *E* > 0 is only weakly perturbed by the moiré potential (Figure 1d and ref (16)) and the level spacing is small at moderate *B* values. Moreover, because the β orbit Fermi surface is much larger (Figure 3b), . This is also supported by the σ_*xx*_ data. Despite the presence of β orbits, the conductivity oscillations due to quantization of the α orbits are still periodic in 1/*B* at any given *n*. The periodicity yields the area of the α orbit Fermi surface , and the α orbit density equals . The energy derivative gives *D*_*α*_(*E*). In section S7, the *n*_*α*_ extracted from the data is shown in Figure S6b and is indeed much smaller than the sum of the density of the α and β orbits. This validates the approximation . Total density *n* at Fermi energy *E* above the mini-CNP is then simply given by the equation , where *n* and *E* are measured from the mini-CNP, *E*_0_ is the energy of the mini-CNP, *v* is the graphene band velocity, *g* = 4 is the valley and spin degeneracy, and *gn*_0_ is the density for full occupation of the first miniband. By defining the inverse function , we can convert the trajectory of a conductivity maximum in the Wannier diagram to the energy of a Landau level . Figure 5 displays the Landau level spectrum extracted from the data in Figure 1a in this manner. Note that we can extract only the energy spacing between adjacent levels and not their absolute energy as the energy of the lowest level is unknown.

**Figure 5:**
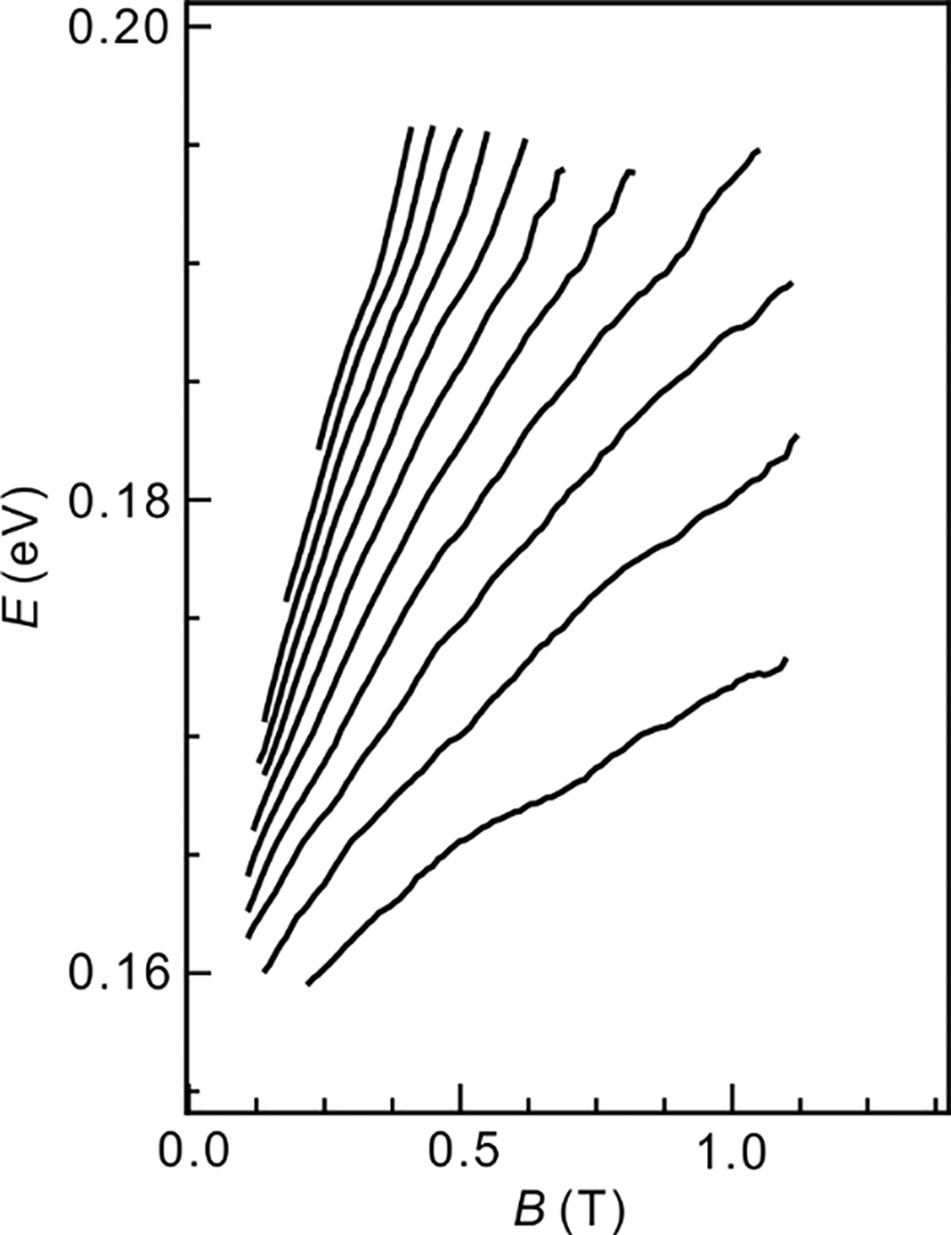
Energy spectrum of α orbits decoded by using eq 2 (see the text) from the peaks in the σ_*xx*_ data (see also Figure S6).

In conclusion, we have identified conductivity oscillations that trace anomalous nonlinear trajectories in a Wannier diagram and do not converge to any miniband edge in an aligned graphene/hBN heterostructure. They appear when multiple Fermi surfaces coexist and when magnetic breakdown creates real space orbits with very different areas. Consistent with our picture, nonlinear trajectories are absent for hole population because minibands do not overlap (section S8). The technique described above should also be helpful for other materials with multiple occupied bands to resolve the magnitude and field dependence of the gaps in the energy spectrum from the Wannier diagram, normally an impossible feat.

## Methods

### Device Fabrication

Device D1 is composed of a graphene monolayer encapsulated on either side with an hBN layer. The doped Si substrate covered with a thermal oxide serves as the back gate. The heterostructure was assembled using a viscoelastic stamp method that has been described in detail previously.^18^ We selected hBN flakes and a graphene flake that had either one very straight edge or two boundaries that form a 120° angle. For the top hBN, such a boundary was then intentionally aligned with the straight boundary of the graphene flake using a motorized *x,y,z* and θ stage. A second moiré potential from the bottom hBN flake was avoided by intentionally placing the bottom hBN with respect to the graphene flake at a twist angle of ≥5°. For the second device, D2, the dry pick-up and transfer process using a PPC/PDMS stamp as described in ref (22) was used to assemble a hBN/graphite/hBN/graphene/hBN/graphite heterostructure. Device D2 was fabricated in vacuum (5 × 10^–4^ mbar) to increase the useable area of the layer stack.^23,24^ To reduce the number of bubbles and wrinkles, both devices were annealed for 30 min at 500 °C in forming gas at a pressure of 150 mbar. For further details of the fabrication procedure, see refs (18) and (21).

### Magnetotransport Measurements

Magnetotransport data for D1 were recorded in a top-loading-into-mixture dilution refrigerator from Oxford Instruments at a base temperature of ∼30 mK. Data for D2 were measured in a dry Physical Property Measurement System from Quantum Design (PPMS Dynacool) for temperatures of ≥1.7 K. The longitudinal resistance and transverse resistance were measured in four terminal configurations using a lock-in technique with an alternating current *I* of 10 nA and a frequency *f* of 17.777 Hz.

## Supporting Information Available

Additional magnetotransport data, estimate of the superlattice period and twist angle for device D1, magnetotransport on device D2, superlattice Brillouin zone, extended energy spectrum and Wannier diagram, effective Hamiltonian, area of the Fermi surface of the α orbit, and absence of nonlinear features for hole population (PDF)
