# Supplementary material for: Nonlinear Landau Fan Diagram for Graphene Electrons Exposed to a Moiré Potential
Source: Nano Lett. 2024 Feb 2;24(11):3339–46. doi: 10.1021/acs.nanolett.3c04444 (PMC10958500; doi:10.1021/acs.nanolett.3c04444)
Supplement: Supplementary file 1 — nl3c04444_si_001.pdf [file nl3c04444_si_001.pdf]

Supporting Information for "Non-linear Landau fan diagram for graphene electrons exposed to a moiré potential"

Pilkyung Moon[1,2,△,*], Youngwook Kim[3,4,△], Mikito Koshino[5,△,*], Takashi Taniguchi[6], Kenji Watanabe[7], and Jurgen H. Smet[3,*]

[1]*Arts and Sciences, NYU Shanghai, Shanghai 200124, China*

[2]*NYU-ECNU Institute of Physics at NYU Shanghai, Shanghai 200062, China*

[3]*Max-Planck-Institut für Festköperforschung, 70569 Stuttgart, Germany*

[4]*Departiment of Physics and Chemistry, DGIST, 42988, Korea*

[5]*Department of Physics, Osaka University, Toyonaka 560-0043, Japan*

[6]*International Center for Materials Nanoarchitectonics, National Institute for Materials Science, Tsukuba 305-0044, Japan*

[7]*Research Center for Functional Materials, National Institute for Materials Science, Tsukuba 305-0044, Japan*

[△]These authors contributed equally

*E-mail: pilkyung.moon@nyu.edu, koshino@phys.sci.osaka-u.ac.jp, and j.smet@fkf.mpg.de

S1. EXTENDED MAGNETOTRANSPORT DATA

The color legends and the maximum magnetic field in the top panels of Fig. 1a and 1b were selected so as to enhance the visibility of the non-linear trajectories traced by the conductivity minima at high electron densities ($V_g > 50$ V), that are central to this work. The complete data set up to 21.5 T recorded on device D1 is shown in Fig. S1 using an appropriately adjusted color legend. The conventional linear Landau fans that emerge from the main and mini charge neutrality points at zero field are better visible in this extended data set. Integer quantum Hall states are observed from 0.5 T and broken symmetry states appear near 3 T. The commensurability between the moiré superlattice unit cell and the magnetic unit cell produces features associated with the fractal Hofstadter energy spectrum. For instance, the horizontal white lines in σ_{xx} signal high conductivity due to the formation of energy bands with a finite width at commensurate fields, where the normalized magnetic flux ϕ/ϕ_0 (ϕ=BA, $\phi_0 = h/e$) takes on a rational number p/q. Here, p and q are coprime integers. They are known as Brown-Zack oscillations [S1-S3]. More than 80 quantum Hall states associated with the fractal spectrum can be identified starting from 2 T in this color map. All of these features are flux linear.

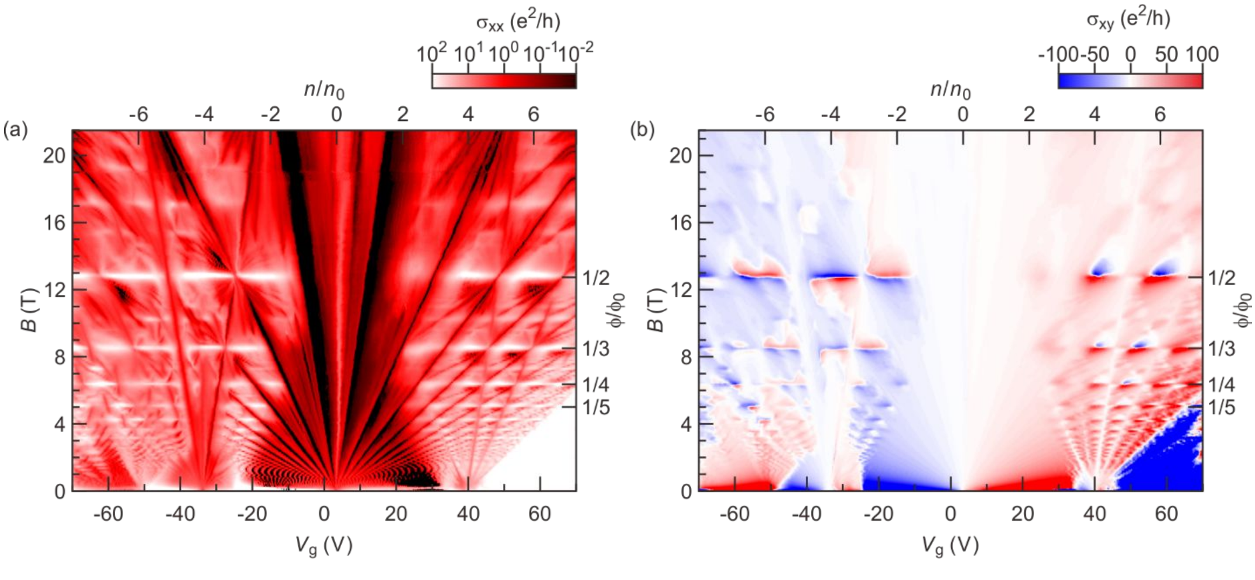

Figure S1. Extended data set for device D1 up to 21.5 T complementing Fig. 1 in the main text. The color legend was adjusted and optimized for improved visibility of the linear Landau fans and the Hofstadter butterfly features. The dimensionless magnetic field, ϕ/ϕ_0, is used as the right ordinate due to its relevance for the fractal butterfly spectrum. Here, ϕ is the flux contained in the moiré superlattice unit cell and ϕ_0 is the magnetic flux quantum, (h/e).

S2. ESTIMATE OF THE SUPERLATTICE PERIOD AND TWIST ANGLE FOR DEVICE D1

The size of the moiré superlattice unit cell or twist angle can be estimated from the density required to go from the main CNP at the Dirac point to the mini CNP when the first miniband is completely filled. For device D1 this requires a change in the gate voltage of approximately 36.2 V. The density at this gate voltage is obtained from an analysis of the low magnetic field Shubnikov de-Haas oscillation frequency or alternatively the filling factor of quantum Hall states at a high magnetic field. It is approximately equal to 2.49 × 10^{12} cm^{-2} and should correspond to $g_s g_v n_0$, where n_0 is the inverse superlattice unit cell area = $[3^{-1/2}/2 \times a^2]^{-1}$. Here, a denotes the lattice constant of the superlattice unit cell and $g_s g_v$ (=4) refers to the internal degrees of freedom due to spin and valley.

The expression yields a superlattice unit cell of approximately 13.9 nm for device D1 or a twist angle between hBN and graphene of 0°±0.1. The Hofstadter butterfly features also allow to identify at what magnetic field a single magnetic flux quantum penetrates the superlattice unit cell. This occurs at a field of approximately 26 T, close to the expected 25.2 T for a zero twist angle graphene/hBN heterostructure.

A second method to determine the superlattice periodicity is the use of Brown-Zak oscillations [S1-S3] in Figs. S1a and S1b that appear at the simplest rational combinations p/q of the normalized flux ϕ/ϕ_0, namely $1/n$ where n is an integer. These quantum oscillations are density independent, since the total flux $\phi = B \times [3^{-1/2}/2 \times a^2]^{-1}$and just depends on the size of the superlattice unit cell. A best fit to the data yields a = 13.7 nm. This value corresponds to a twist angle of 0.15°±0.05.

There is another alternative way to estimate the size of the superlattice unit cell using the Diophantine relation. It is described in Section S3 below.

S3. MAGNETOTRANSPORT ON DEVICE D2

Device D2 consists of a hBN/graphene/hBN heterostructure sandwiched between both a top and bottom graphite gate. Since we are working with monolayer graphene, the application of an

additional top-gate voltage provides no new information, since the response of the sample induced by the top-gate is identical to that of the back-gate. Even if so, there can be advantages to include a top-gate in monolayer graphene devices. First, it offers the ability to populate the graphene layer up to larger charge carrier density. However, for the phenomenon addressed here, there is no benefit, since it is observable within the charge carrier window accessible with a single gate. Second, the conducting top-gate can help to improve the overall transport quality [S4] by screening fluctuations of the disorder potential induced for instance by adsorbates on the topmost surface. In our case, this was the primary reason to incorporate a graphite top-gate in device D2 as well as the graphite-based bottom gate. For recording the data on device D2, the top-gate was either left floating or connected to ground (no difference). It was not used for conventional gating purposes.

Fig. S2a shows the dependence of the longitudinal resistance on the back-gate voltage at T = 1.7 K and B = 0 T. The resistance exhibits a sharp maximum at 0 V corresponding to the main CNP as well as two satellite peaks near ± 6 V when the lowest miniband is completely filled or emptied. A very rich sequence of quantum Hall states due to the Hofstdter butterfly spectrum can be clearly seen. The field and density coordinates, where these states appear, obey the Diophantine equation: $(n/n_0) = t\times(\phi/\phi_0) + s$. Here, t is the Chern number and s represents the index for Bloch band filling. The periodicity of the moiré superlattice period can be obtained from the crossing of the fan line with $t = -6$ from the mini CNP on the electron side and the fan line with $t = 6$ from the charge neutrality point. These two quantum Hall states cross at B = 12 T as shown in Figure S2b. From the Diophantine relation, we can construct the following relation, $(n/n_0) = 6(\phi/\phi_0) = -6(\phi/\phi_0) + 4$, hence $\phi/\phi_0 = 1/3 = B \times$ (unit cell area) $\times\ e/h$. This implies that the superlattice periodicity, a, equals 12.3 nm. This corresponds to a twist angle of 0.6º between hBN and graphene.

Figure S2c shows a data set recorded for a smaller range of magnetic fields between -3 T and +3 T in the regime of large electron densities. In the vicinity of V_g = 6 V, multiple quantum Hall states appear near the mini-CNP. At even higher densities, the resistance shows multiple minima that follow non-linear trajectories. Two of these are highlighted with white solid lines. To emphasize the non-linearity, white dashed lines are included that are linear extrapolations of these two resistance minima from high field down to zero field. The non-linear features behave similar as in

device D1. Their strength weakens with increasing magnetic field and they finally disappear near 3 T.

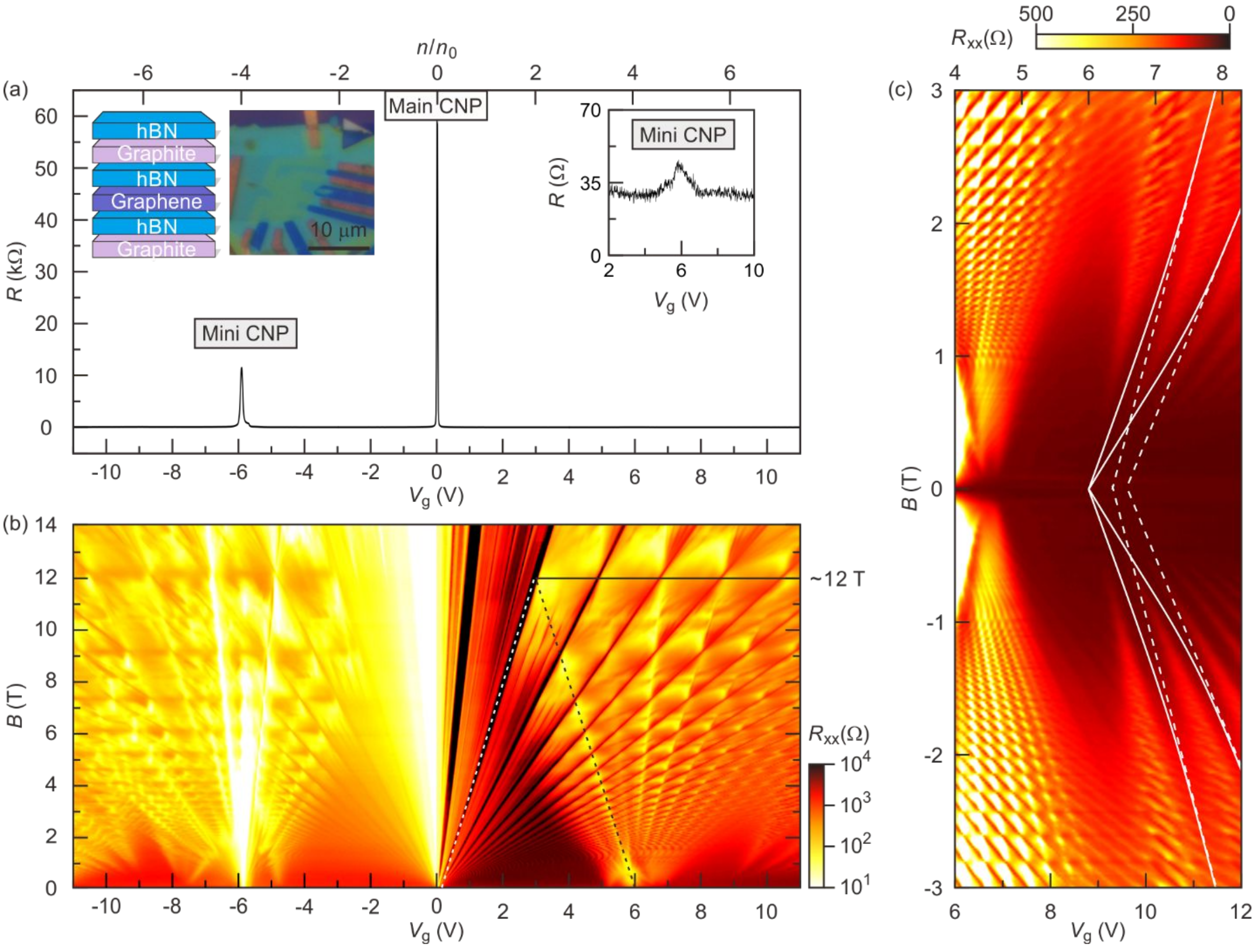

Figure S2. Magnetoresistance data recorded on device D2. (a) Longitudinal resistance R_{xx} as a function of back-gate voltage and n/n_0 for B = 0T. The resistance peaks when the highest valence miniband is completely emptied of electrons, the lowest conduction miniband is completely occupied with electrons or when the chemical potential crosses the Dirac point. Left inset: Schematic of the device structure as well as an optical image. The scale bar equals 10 μm. Right inset: Enlargement of the weak resistance peak when the lowest conduction miniband gets fully occupied. (b) Longitudinal magnetoresistance as a function of field and density for fields up to 14 T. The data set is dominated by Hofstadter butterfly features. The white dashed line marks the quantum Hall feature for t = 6 in the Diophantine equation. It emanates from the main charge neutrality point. The black dashed line corresponds to the quantum Hall feature for t = -6. It emerges from the mini CNP of the lowest conduction miniband instead. These lines cross near 12 T (black horizontal line). (c) Magnetoresistance as a function back-gate voltage V_g and field B for a smaller range of magnetic fields and the regime of high electron density. The field is swept from −3 T to +3 T in 10 mT step, whereas the back-gate voltage is varied from 6 V to 12 V in 1 mV

steps. Two of the resistance minima that follow a non-linear trajectory are highlighted with solid white lines. The dashed lines are linear extrapolations of the position of these resistance minima at high fields.

S4. SUPERLATTICE BRILLOUIN ZONE

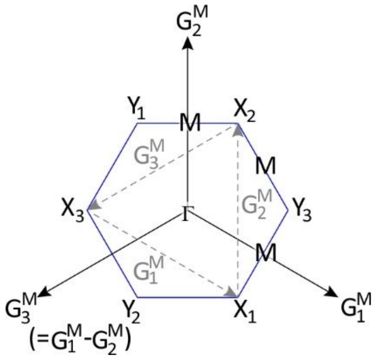

Figure S3. Superlattice Brillouin zone of the graphene/hBN moiré superlattice spanned by the reciprocal lattice vectors $\mathbf{G}_i^M$. X_i and Y_i (i = 1, 2, 3) denote the two inequivalent corners of the superlattice Brillouin zone, aka mini CNP point. Note that $\mathbf{G}_i^M$ connects the three equivalent corners X_i (and Y_i as well). Thus, the moiré superlattice potential couples the Dirac spinors at these corners.

S5. EXTENDED ENERGY SPECTRUM AND WANNIER DIAGRAM

Figures S4a and S4b show the plots similar to Figs. 2c and 2d but for a wider range of electron energy and density. Both figures clearly show the linear trajectories of the minima in the density of states that converge to the band edges at $n/n_0 = 0, 4$ as well as the non-linear trajectories starting from $n/n_0 > 5$.

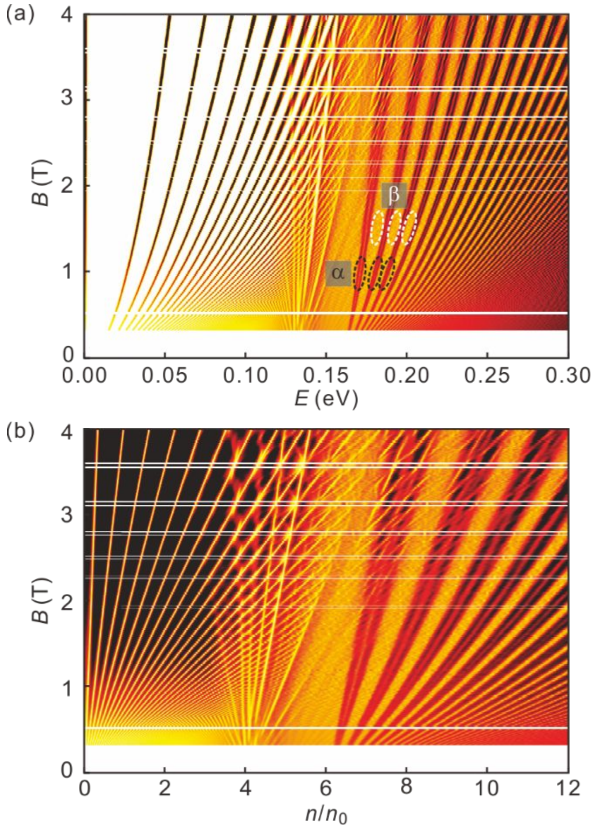

Figure S4. Plot of the density of states across of the (B,E) and $(B,n/n_0)$ parameter space as in Figs. 2c and 2d of the main text, but for a wide range of electron energies and densities. Darker color corresponds to a large DOS. The quantization of the α-orbits is responsible for the enhanced density of states in regions such as those marked by the dotted blank lines in panel a. The β orbits are responsible for the bright orange background as seen in the regions demarcated by the white dotted lines.

S6. EFFECTIVE HAMILTONIAN

In the main text and Fig. 3, we attribute the presence of two different series of Landau levels associated with the α- and β-orbits to the strong asymmetry in the energy dispersions near the X and Y corners of the reduced Brillouin zone. The isoenergetic contours of the third conduction miniband near the X corners are well separated in reciprocal space from the isoenergetic contours of the other minibands. This gives rise to a discrete set of Landau levels. However, isoenergetic contours of the third conduction miniband near the Y corner nearly touch the isoenergetic contours of the second conduction miniband, giving rise to an almost continuous energy spectrum. This distinction between the contours near X and Y originates from the different coupling strength at

these corners. The Hamiltonian around the zone corners Z_i^η ($i = 1,2,3$) (Z_i^η is X_i for $\eta = 1$ and Y_i for $\eta = -1$) near the K-valley of monolayer graphene can be written as

$$H = IE_0^Z + \begin{pmatrix} s\hbar v\tilde{k}\cos\left(\theta + \frac{\pi}{3}\right) & \alpha^* + \beta^*\tilde{k}\cos\theta & \alpha + \beta\tilde{k}\cos\left(\theta + \frac{2\pi}{3}\right) \\ \alpha + \beta\tilde{k}\cos\theta & s\hbar v\tilde{k}\cos\left(\theta - \frac{\pi}{3}\right) & \alpha^* + \beta^* k\cos\left(\theta - \frac{2\pi}{3}\right) \\ \alpha^* + \beta^*\tilde{k}\cos\left(\theta + \frac{2\pi}{3}\right) & \alpha + \beta k\cos\left(\theta - \frac{2\pi}{3}\right) & -s\hbar v\tilde{k}\cos\theta \end{pmatrix} \quad \text{(S1)}$$

in the basis of $|Z_1^\eta + \tilde{\boldsymbol{k}}\rangle$,$|Z_2^\eta + \tilde{\boldsymbol{k}}\rangle$,$|Z_3^\eta + \tilde{\boldsymbol{k}}\rangle$ and up to the first order in $\tilde{k}$. Here, $|\boldsymbol{k}\rangle$ is the wave function of unproximitized graphene for the vector $\boldsymbol{k}$, where $\tilde{\boldsymbol{k}} = \tilde{k}(\cos\theta, \sin\theta)$ is the wave vector measured from each Z_i^η, $E_0^Z = s\hbar v G^M/\sqrt{3}$ is the energy of unproximitized graphene at Z_i^η, s is 1 for the conduction band and -1 for the valence band, v is the Fermi velocity of monolayer graphene, $G^M = |G_i^M|$, I is the 3×3 identity matrix, and

$$(\alpha,\beta) = \begin{cases} V_1 e^{i\eta\psi}(\omega^*/2, (-\omega + 2\omega^*)\gamma) & (s = 1, \eta = 1) \\ (0, 0) & (s = 1, \eta = -1) \\ V_1 e^{i\eta\psi}(-3\omega^*/2, 3\omega\gamma) & (s = -1, \eta = 1) \\ V_1 e^{i\eta\psi}(2, 2(1 - 2\omega^*)\gamma) & (s = -1, \eta = -1) \end{cases}, \quad \text{(S2)}$$

where $V_1 \approx 0.0210$ eV, $\psi \approx -0.29$ (rad), and $\gamma = \sqrt{3}/2G^M$. A similarity transformation of H with

$$U = \frac{1}{\sqrt{3}}\begin{pmatrix} 1 & 1 & 1 \\ 1 & \omega & \omega^* \\ 1 & \omega^* & \omega \end{pmatrix} \quad \text{(S3)}$$

gives

$$H' = U^\dagger H U = IE_0^Z + \begin{pmatrix} \alpha + \alpha^* & -\frac{\omega^*}{2}(s\hbar v - \beta^*\omega^* - \beta\omega)\tilde{k}e^{i\theta} & -\frac{\omega}{2}(s\hbar v - \beta\omega^* - \beta^*\omega)\tilde{k}e^{-i\theta} \\ -\frac{\omega}{2}(s\hbar v - \beta\omega - \beta^*\omega^*)\tilde{k}e^{-i\theta} & \alpha\omega^* + \alpha^*\omega & -\frac{\omega^*}{2}(s\hbar v - \beta^* - \beta)\tilde{k}e^{i\theta} \\ -\frac{\omega^*}{2}(s\hbar v - \beta^*\omega - \beta\omega^*)\tilde{k}e^{i\theta} & -\frac{\omega}{2}(s\hbar v - \beta - \beta^*)\tilde{k}e^{-i\theta} & \alpha\omega + \alpha^*\omega^* \end{pmatrix}$$

. (S4)

The bottom panels in Fig. 3a show the energy dispersion of the conduction bands (s = 1) plotted against $\tilde{\boldsymbol{k}}$ near X (η = 1) and Y (η = −1), respectively. At the zone corner ($\tilde{k} = 0$), the eigenvalues are $E = \{E_0^Z + \alpha + \alpha^*, E_0^Z + \alpha\omega^* + \alpha^*\omega, E_0^Z + \alpha\omega + \alpha^*\omega^*\} = \{E_0^Z + CV_1\cos\psi, E_0^Z + CV_1\cos(\psi + 2\pi/3), E_0^Z + CV_1\cos(\psi - 2\pi/3)\}$, where C is 1 (−3) and 0 (4) for X and Y in the conduction (valence) bands, respectively. Thus, similar to the numerical calculation of the band structures (top panel in Fig. 3a), the first three conduction bands at Y are degenerate, while the bands at X ($\{E_0^Z - 0.015, E_0^Z - 0.005, E_0^Z + 0.220\}$), especially the third band, are split by a finite amount. Thus, the electron pocket of the third band at Y tunnels to the second band and is hybridized to a band with very large area S, while the pocket encircling X works as an almost independent band with small S. To be more precise, the interaction that is of higher order in $\mathbf{G}_i^M$ opens a tiny pseudogap at this point, but this small opening does not prevent the band hybridization discussed in the main text.

The dispersion of the valence minibands ($s = -1$) in Fig. S5 shows that these bands are well separated in energy by the large band gap at the mini CNP points, unlike what happens to the conduction minibands (Fig. 3a).

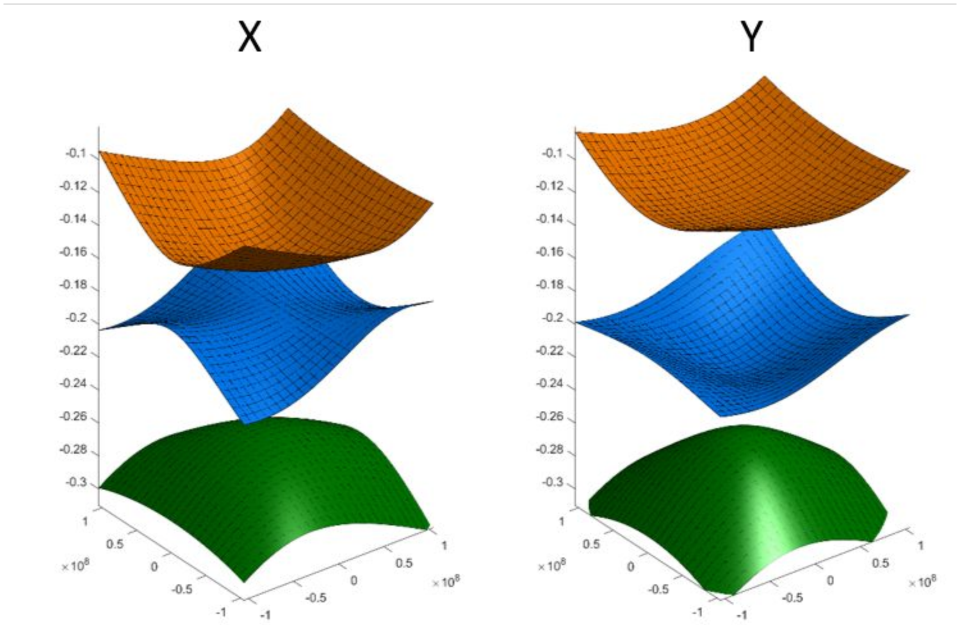

Figure S5. Dispersion near the X (left) and Y (right) corners of the three topmost valence minibands, calculated by the effective model (Eq. S4). The minibands are well separated and do not overlap.

S7. AREA OF THE FERMI SURFACE OF THE ALPHA-ORBIT

As described in the main text, it is not necessary to have any information about D_α in order to extract the energy difference between two adjacent α-orbit Landau levels, $\Delta E_i{}^\alpha$. Instead, we need to have some information about D_β. This follows immediately from the 2nd term of Eq. (1) that contains $\Delta E_i{}^\alpha$ as well as D_β, but nothing else. In the graphene/hBN heterostructure, $D_\alpha + D_\beta$ follows the density of states of monolayer graphene, since the dispersion for electron population ($E_F > 0$) is only weakly modified by the moiré potential compared with the linear dispersion of an unproximitized graphene monolayer (Fig. 1c, main text). In addition, it can be argued that $D_\alpha \ll D_\beta$, so that $D_\alpha + D_\beta \approx D_\beta$. This assertion is justified based on the large difference between the two Fermi surface areas associated with the α- and the β- orbits (Fig. 3b), since the Fermi surface area divided by $4\pi^2$ yields the electron density and the derivative of the electron density with respect to energy in turn equals the density of states. Importantly, this assertion is supported here by the σ_{xx} -data. At a fixed sample density, the peaks in σ_{xx} caused by the quantization of the α-orbit are still periodic with 1/B just as for conventional Shubnikov-de Haas oscillations and the 1/B periodicity is a direct measure of the α-orbit Fermi surface area. In the experimental data in Fig. S6a, the black lines mark the conductivity features associated with the quantization of the α-orbit. For each density, the 1/B periodicity of these features is plotted in Fig. S6b (red line). The extracted values are consistent with the theoretically expected behavior (blue line). The total density of electrons located in the second and the third conduction miniband is also shown as the black dashed line. It corresponds to $D_\alpha + D_\beta$ and does not include the density of the electrons occupying the completely filled lowest conduction miniband that hosts a density of $4n_0$. Clearly the number of electrons occupying the α-orbit Fermi surface pocket is small and indeed $D_\alpha \ll D_\beta$ or $D_\alpha + D_\beta \approx D_\beta$. With this single approximation for D_β it is possible to obtain the energy differences between adjacent α-orbit Landau levels, $\Delta E_i{}^\alpha$, using Eq. (1) of the main text.

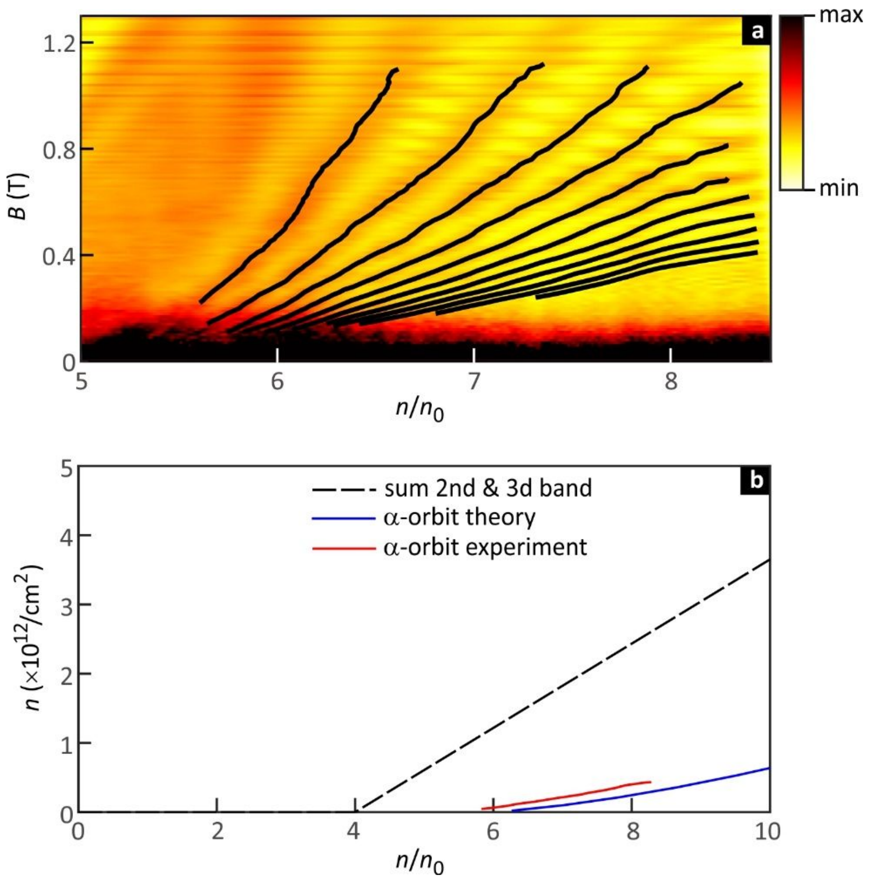

Figure S6. (a) σ_{xx} measured in experiment and plotted in the n-B plane. Black solid lines trace the Landau levels from the α-orbit. (b) Plot of the electron density occupying α-orbit states as determined from theory (blue) and an analysis of the 1/B periodicity of the conductivity oscillations observed in experiment (red). The theoretical trace is consistent with the experimental data. Also plotted is the density of electrons occupying either α- or β-orbits (dashed black line). This density is equal to the total density of electrons occupying the second and third conduction miniband. It also corresponds to the electron density in a pristine graphene monolayer not subjected to the moiré potential after subtracting the electron density of the first electron superlattice miniband ($4n_0$). Clearly, the electron density executing α-orbits is much smaller than the density of electrons tracing β-orbits.

For the sake of completeness, we note that $D_\alpha \ll D_\beta$ will always be true in a system with two sets of Landau levels α and β with a significantly different spacing between the levels, since this spacing is inversely proportional to the Fermi surface area, at least as long as the number of miniband pockets for α and β are not very different. In a system where the number of miniband

pockets for the orbit with the larger level spacing (i.e., smaller Fermi surface and smaller electron density) is extremely large, the electron densities for α and β might end up being comparable, but that should be an extraordinary rare and coincidental case.

S8. ABSENCE OF NON-LINEAR FEATURES FOR HOLE POPULATION

For electron population, the measured magnetotransport data as well as the theoretically calculated density of states exhibit a non-linear fan of features, because of the overlap on the energy axis between the second and the third miniband. The corresponding regions are marked with a box, that is demarcated with a dashed white line and labeled as box 1 in Fig. S7a and Fig. S7b. The overlap of the minibands can easily be identified in the plot of the dispersion of the minibands in Fig. S7c. The second miniband is displayed in orange and the third miniband in blue. For hole population such non-linear behavior is not observed (boxes demarcated with solid lines and labeled as box 2 in Fig. S7a and Fig. S7b), because the energy overlap between the first and second valence miniband as well as between the second and third valence minibands is small. This is seen nicely in Fig. S7c and Section 6 in SI.

However, it should be noted that the fan diagrams for a hole population away from n/n_0 – 4 or – 8 also becomes quite complicated as soon as n/n_0 deviates from −8 or −4 because of (i) the van Hove singularity as well as (ii) the lift of the valley degeneracy. (i) The van Hove singularity is the energy where the band topology changes from an electron-like dispersion, which increases the Fermi surface as the electron energy increases, to a hole-like dispersion, which decreases the Fermi surface as the energy increases. There, the electron-like Landau levels and hole-like Landau levels meet, and yields multiple maxima to the density of states as magnetic field increases ($n/n_0 \sim -6.5$ and −3.5 in Fig. S7b, $E \sim -0.18$ eV and −0.10 eV in Fig. S7d). This feature is common to the Landau levels in any other systems. (ii) The solid and dotted lines in Fig. S7c represent the bands near the K and K' valley of monolayer graphene. They are degenerate in energy in the absence of a magnetic field but their Landau levels are not, since the graphene/hBN heterostructure lacks inversion symmetry. The lift of the degeneracy is quite pronounced for the second valence miniband, thus the two sets of Landau levels cross at multiple points in the E-B plane ($E \sim -0.18$ eV in Fig. S7d). This yields a complicated sequence of maxima in the density of states ($n/n_0 \sim -6.5$ in Fig. S7b).

Please note that the lift of the degeneracy between the Landau levels from the *K* and *K′* valleys of monolayer graphene doubles the number of fans and halves the degeneracy of each fan, but does not disturb the linear behaviour of the Landau fans near $n/n_0 = -8$ or -4 as is obvious from Figs. 4a-4c.

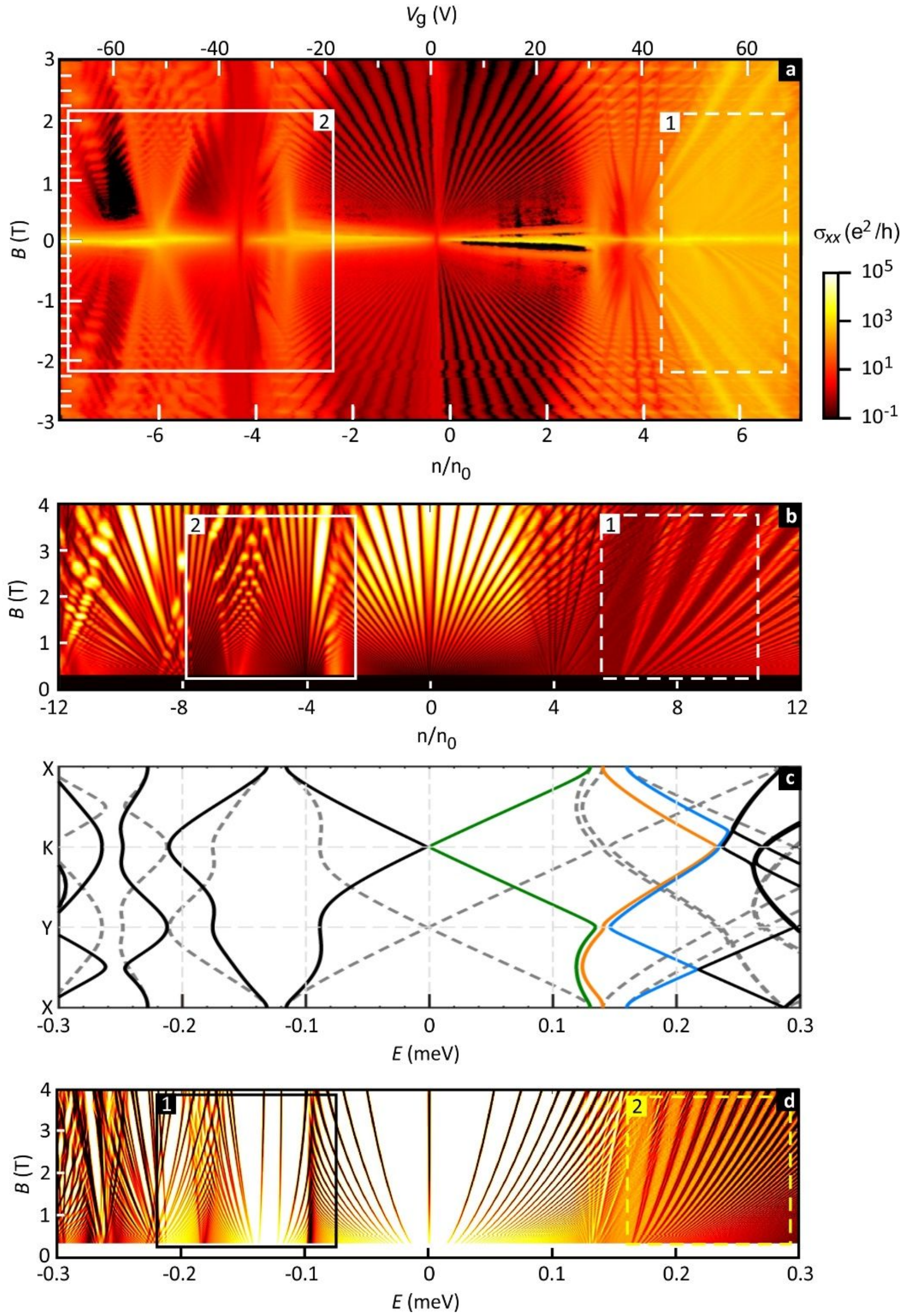

Figure S7. (a) Replot of Fig. 1a of the main text: σ_{xx} of a graphene/hBN heterostructure with a 0° twist angle measured at 30 mK as a function of gate voltage (V_g) or normalized density (n/n_0) and

magnetic field (*B*). A logarithmic color scale is used. The magnetic field covers the range -3T to +3T. (b) Theoretically calculated density of states in the *n* versus *B* plane. The scale and range of *n* are set equal to those in (a). (c) Replot of Fig. 1c of the main text: band dispersions along the high symmetry points of the Brillouin zone (see Fig. S3). Solid (dotted) lines represent the bands near the *K* (*K*') valley of monolayer graphene. (d) Theoretically calculated density of states in the *E* versus *B* plane. The scale and range of *E* are set equal to those used in panel (c).

Reference for Supporting Information

[S1] Brown, E. "Bloch electrons in a uniform magnetic field." *Phys. Rev*. **1964** 133, A1038.

[S2] Zak, J. "Magnetic translation group." Phys. Rev. **1964** 134, A1602.

[S3] Krishna Kumar; R., Chen; X., Auton; G. H., Mishchenko; A., Bandurin; D. A., Morozov; S. V., Cao; Y., Khestanova; E., Ben Shalom; M., Kretinin; A. V., Novoselov; K. S., Eaves; L., Grigorieva; I. V., Ponomarenko; L. A., Fal'ko; V. I., and Geim; A. K., "High-temperature quantumoscillations caused by recurringBloch states in graphene superlattices" Science **2017** 357, 181-184.

[S4] Zibrov; A. A., Kometter; C., Zhou; H., Spanton; E. M., Taniguchi; T., Watanabe; K., Zaletel; M. P., and Young; A. F., "Tunable interacting composite fermion phases in a half-filled bilayer-graphene Landau level" *Nature* **2017**, 549, 360-634.
